# Supplementary material for: Splice-Junction-Based Mapping of Alternative Isoforms in the Human Proteome
Source: Cell Rep. Author manuscript; Available in PMC 2020 Jan 15. (PMC6961840; doi:10.1016/j.celrep.2019.11.026)
Supplement: 2 [file NIHMS1546469-supplement-2.zip › DF1/PXD010154/Prostate/Prostate_8_TRIM28_AMCSLTAAAASAASGSPGPGEGSAGGEKR.pdf]

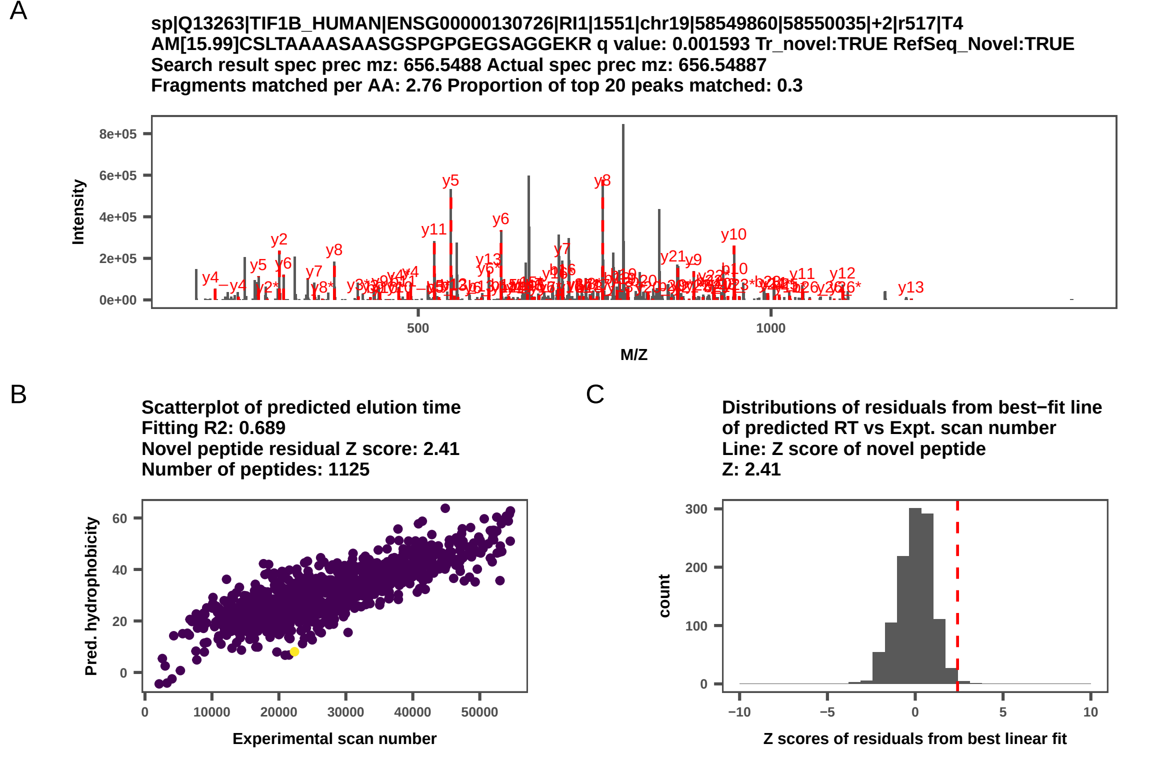
A
sp|Q13263|TIF1B_HUMAN|ENSG00000130726|RI1|1551|chr19|58549860|58550035|+2|r517|T4
AM[15.99]CSLTAAAASAASGSPGPGEGSAGGEKR q value: 0.001593 Tr_novel:TRUE RefSeq_Novel:TRUE
Search result spec prec mz: 656.5488 Actual spec prec mz: 656.54887
Fragments matched per AA: 2.76 Proportion of top 20 peaks matched: 0.3
Intensity
8e+05
6e+05
4e+05
2e+05
0e+00
y5
y8
y6
y11
y10
y2
y8
y7
y4
y5
y6
y13
y21
y9
b10
y11
y12
y13
500
1000
M/Z
B
Scatterplot of predicted elution time
Fitting R2: 0.689
Novel peptide residual Z score: 2.41
Number of peptides: 1125
Pred. hydrophobicity
60
40
20
0
0
10000
20000
30000
40000
50000
Experimental scan number
C
Distributions of residuals from best−fit line
of predicted RT vs Expt. scan number
Line: Z score of novel peptide
Z: 2.41
count
300
200
100
0
−10
−5
0
5
10
Z scores of residuals from best linear fit
